# Abnormal oligodendrocyte function in schizophrenia explains the long latent interval in some patients

**DOI:** 10.1038/s41398-022-01879-0

**Published:** 2022-03-25

**Authors:** Jeffrey Fessel

**Affiliations:** Department of Medicine, University of California, San Francisco, CA USA

**Keywords:** Pathogenesis, Clinical genetics

## Abstract

A puzzling feature of schizophrenia, is the long latency between the beginning of neuropathological changes and the clinical presentation that may be two decades later. Abnormalities in oligodendrocyte function may explain this latency, because mature oligodendrocytes produce myelination, and if myelination were abnormal from the outset, it would cause the synaptic dysfunction and abnormal neural tracts that are underpinning features of schizophrenia. The hypothesis is that latency is caused by events that occur in some patients as early as in-utero or infancy, because clones of abnormal, myelinating oligodendrocytes may arise at that time; their number doubles every ~2 years, so their geometric increase between birth and age twenty, when clinical presentation occurs, is about 1000-fold plus the effect of compounding. For those patients in particular, the long latency is because of a small but ongoing increase in volume of the resulting, abnormally myelinated neural tracts until, after a long latent interval, a critical mass is reached that allows the full clinical features of schizophrenia. During latency, there may be behavioral aberrancies because of abnormally myelinated neural tracts but they are insufficiently numerous for the clinical syndrome. The occurrence of behavioral symptoms during the long latent period, substantiates the hypothesis that abnormal oligodendrocytes explain the latency in some patients. Treatment with fingolimod or siponimod benefits both oligodendrocytes and neural tracts. Clinical trial would validate their potential benefit in appropriate patients with schizophrenia and, concurrently, would validate the hypothesis.

## Introduction: General overview, abnormal oligodendrocytes in schizophrenia, childhood stressors, effects of stressors on oligodendrocytes, latent interval

Many have written about the profiles of individuals who are at high risk for developing subsequent psychosis; but no one has explained the long latency of 20+ years between the early events causing neuropathological changes and the much later, clinical presentation of schizophrenia (SCZ). A long latency affects many patients, particularly those with inciting events either in-utero or infancy that may trigger the start of neuropathology. The hypothesis proposed here is that, for those patients in particular, anatomical and functional abnormalities that commence in myelinating oligodendrocytes at the time of the inciting events, may explain the latency. If correct, a practical consequence of this hypothesis is that fingolimod treatment benefits myelinating oligodendrocytes (see below for details) and potentially may benefit such patients.

The background of the hypothesis is as follows. First, a critical function of mature oligodendrocytes is myelination, and if oligodendrocytes have abnormal function, they would cause synaptic dysfunction and abnormal neural tracts, which are anatomical features of SCZ. It is postulated that latency sometimes occurs because, in SCZ, abnormal oligodendrocytes may develop as early as in-utero or infancy; and since their doubling time is about 2 years, their geometric increase between birth and age twenty, which is the approximate age when SCZ becomes overt, is about 1000-fold even without accounting for the effect of compounding. Latency occurs because abnormally functioning, myelinating oligodendrocytes create a continuous, small but progressive, increase in the volume of abnormal neural tracts until when those tracts reach a critical mass, the full clinical features of SCZ emerge. During that latent interval there may be minor behavioral aberrancies, because the abnormal neural tracts are still insufficiently numerous for the clinical syndrome.

### Myelination and oligodendrocytes, and their doubling time

Myelination by oligodendrocytes is an ongoing process between infancy and adulthood, and abnormal neural tracts result from abnormalities of the myelinating oligodendrocytes. A pool of undifferentiated oligodendrocyte precursor cells (OPC) remains in the parenchyma of the adult CNS, and they can differentiate into pre-myelinating and myelinating, mature oligodendrocytes [1]. Abnormal forms are detectable in SCZ: Vikhreva et al. reported that 21 patients with SCX had degenerative changes in oligodendrocytes that included swelling, vacuolation, paucity of ribosomes, reduction in volume and number of mitochondria, and an increase in lipofuscin granules [2]. Mauney et al. saw results in SCZ showing impairment of OPC differentiation into mature oligodendrocytes, that contributed to myelin deficits [3].

Studies show that the doubling time of oligodendrocytes is ~2 years. Tripathi et al. tracked the survival in mice, of mature, myelinating oligodendrocytes in the optic nerve, where myelinating oligodendrocytes had a half-life ~2 years [4]. Hill et al. also showed survival of oligodendrocytes as ~2 years, by using live imaging in layer I of the mouse cerebral cortex [5]. If one abnormal clone renews every 2 years between ages 0 and 20, it would multiply ~1000-fold by geometric progression, and yet more by compounding; in that way, a single abnormal clone at birth or infancy, would quickly become dominant. Yeung et al. measured myelin volume in the human postmortem corpus callosum from 57 subjects aged 0.2–92 years; the myelin volume reached its maximum at age 17 years [6], which is the approximate age when symptomatic SCX often first appears but it is often later, in the mid-third decade of life. Analyses of scans showing fractional anisotropy (FA) that reflects myelin (see section below, ‘Imaging and Myelination’), in 600 patients with SCZ and 492 healthy controls, indicated that myelination reached full maturation at age 27 years in the patients and 33 years in the controls [7]. It is a plausible hypothesis that clinical manifestations of SCZ appear between ages 17 and 27 at a point in time when there is now an adequate number of tracts with abnormal myelination and synapses.

### Stressors are deleterious for the oligodendrocyte lineage

Many reports show that stressors of various sorts affect oligodendrocyte maturation and myelination and, thus, impact neuronal function and connectivity. Makinodan et al housed mice under one of the following conditions beginning at weaning on postnatal day 21: in isolation with a single mouse per cage; or in either a regular or enriched environment with four or eight mice per cage, and novel toys [8]. In their prefrontal cortex (PFC), the isolated mice as compared with the others, had oligodendrocytes with shorter processes that had less branching; fewer myelin genes MBP and MAG; and electron microscopy showed reduced myelin thickness. Liu et al. subjected mice to three different stressors, and showed reduction of both myelin and oligodendrocyte gene transcripts [9]. Lehmann et al. induced chronic social stress by co-housing an intruder male mouse in the home cage of a dominant aggressor male mouse, separating the pair with a perforated partition [10]. Each day, the partition was removed for 5 min to allow aggressive encounters between the pair. Then, after 14 days, gene expression changes were assessed, and showed that 69% of the significantly down-regulated genes were myelin-related. Liu et al. caused isolation stress by housing mice singly for a duration of 2–8 weeks and compared them with mice that were housed with five mice per cage [11]. The isolation caused decreased myelin in the PFC associated with the presence of oligodendrocytes that had immature nuclear chromatin and a lower proportion of nuclear heterochromatin. Banasr et al subjected mice to a dozen different stressors, which resulted in a 39% reduction of oligodendrocytes [12]. Finally, Xu et al. subjected mice to social defeat stress and, although they did not see a reduction in oligodendrocytes, the level of MBP protein in the frontal cortex was significantly lowered [13]. In addition, oligodendrocytes are particularly sensitive to glutathione depletion, which is an important defense against the reactive oxygen species (ROS) that rise with stress [14]. Back et al. induced decreased glutathione levels in both OPCs and oligodendrocytes, which completely killed the OPCs although mature oligodendrocytes remained viable [15].

Demonstrating that, also in humans, the stress of childhood trauma correlates with alterations in neural tracts, Eluvathingal et al. reported that the FA values (see section below, ‘Imaging and Myelination’) in the left uncinate fasciculus were decreased in seven children who had had early, severe socio-emotional deprivation in Eastern European orphanages, where neglect was rampant [16]. The inference is that childhood neglect had caused abnormalities in myelinating oligodendrocytes which caused permanently abnormal neural tracts.

### Molecular mechanisms underlying the abnormalities in oligodendrocytes caused by stressors

Molecular mechanisms for the abnormal myelination by oligodendrocytes, include the formation of ROS, and effects of NRG-1/ErbB4, Lingo-1, and Olig2.

Formation of ROS, initiated by persistent endoplasmic reticulum stress and protein misfolding, causes degeneration of oligodendrocytes [17, 18]. In the PFC of the SCZ brain, Prabakaran et al. demonstrated upregulated gene pathways that lead to increased generation of ROS and oxidative stress [19]; and increased generation of ROS was reported by Jiang et al. in the cerebral cortex of socially isolated mice [20].

SCZ is highly heritable, and those factors that identify individuals who are at high risk for subsequent psychosis include, presumably, developmental traits. Neuroregulin 1 (NRG-1) is such a factor, and it also participates in the formation and progression of the oligodendrocyte lineage [21, 22]. NRG-1 identified individuals at high risk for SCZ in two separate cohorts involving 37 persons: Hall et al. and Keri et al. each found that the T/T genotype of the NRG-1 variant SNP8NRG243177 was associated with a 100% rate of transition to psychosis [23, 24]. Interestingly, the level of total NRG-1 in SCZ brain is the same as in controls but the percentage is increased of its isoform II, also known as glial growth factor [25]. The receptor for NRG-1 is ErbB4, whose downstream effects were found by Sussman et al. to suppress maturation of the oligodendrocyte lineage [26]; and the interaction of NRG-1 with ErbB4 was found by Norton et al. to increase susceptibility to SCZ [27]. Law et al. noted that ErbB4 has two isoforms, one of which is ErbB4 CYT-1 that includes a phosphoinositide3-kinase (PI3K) binding site; but although PI3K signaling mediates cell survival, Law et al. observed that in patients with SCZ, ErbB4 has subunits that catalyze, i.e., diminish, PI3P instead of inducing it [28]. In brief, a variant of NRG-1 is a developmental trait that is a risk factor for SCZ, and a diminished NRG1-ErbB4-PI3K signaling system decreases the formation of myelinating oligodendrocytes.

Another negative regulator of OPC differentiation and myelination is LINGO-1 (leucine rich repeat and immunoglobin-like domain-containing protein-1 [29]), whose overexpression caused inhibition of oligodendrocyte differentiation and myelination [30]; and it was significantly (*p* < 0.001) increased in the DLPFC of SCZ brain [31].

Olig2 is a protein that promotes oligodendrocyte differentiation, one mechanism for which is suppression of neuroregulins [32]. Some investigators but not all, have found reductions of Olig2 in SCZ brain, which would reduce numbers of myelinating oligodendrocytes [33].

The hypothesis proposed here is that in those patients who had significant events in their prenatal or immediately postnatal life (see following section), one or more of the above molecular mechanisms may induce an abnormal clone of myelinating oligodendrocytes that commences the lengthy journey to eventual SCZ.

### Prenatal, neonatal, or childhood stressors, and subsequent schizophrenia

The importance of stressors that apply prenatally, neonatally, or in childhood, for causing abnormal oligodendrocytes and later SCZ, is accorded a separate section because the past presence of these particular stressors identifies some of the patients to which the hypothesis may apply. The “dual-hit” hypothesis postulates that an initial event, in this case exposure to a prenatal, neonatal, or childhood stressor, makes an individual susceptible so that a later event, e.g., inflammation, a further stressor, etc., may then be adequate for SCZ to occur. Varese et al reviewed 41 studies and found evidence that childhood adversity was present in 33% of patients with SCZ [34]. The odds ratio (OR) for psychosis was 2.78 for overall adversity, 3.40 for emotional abuse, 2.95 for physical abuse, 2.90 for neglect, 2.39 for bullying, 2.38 for sexual abuse, and 1.70 for parental death. In a notable study, Tienari et al. examined hospital records of 19,447 women who had been in Finnish psychiatric hospitals between 1960 and 1979, and listed those who had been diagnosed with SCZ or paranoid psychosis [35]. They checked every census and parish register throughout Finland, to find those mothers who had sent one or more offspring for adoption; then they matched those offspring and their adoptive families with control families who had adopted offspring of mothers without known SCZ. Psychiatrists interviewed 345 adoptive families in their homes and rated them, based upon 24 specially constructed subscales, for disordered family functioning that might be traumatic for the adoptees. 145 adoptees were at genetic risk because of having biological mothers with SCZ; and 158 other adoptees had mothers with a psychiatric diagnosis that was not SCZ or else a non-psychiatric diagnosis. A median of 23 years later, all adoptees were interviewed by a psychiatrist who was blind as to the status of the biological parents. Diagnoses of SCZ had been made in 40 adoptees, 32 (22.1%) of the 145 who were at genetic risk and only 8 (5.0%) who had no genetic risk, Thus, a genetic risk for SCZ gave an over four-fold-increased risk of developing SCZ in adult life. Notably, adoptees with genetic risk who were reared in good-functioning families had significantly fewer outcomes of SCZ than had adoptees with genetic risk who were reared in worse-functioning families. This important study showed that the parental risk for SCZ had an adverse effect upon the offspring’s environment; it showed the risk conferred by childhood trauma upon the later occurrence of SCZ; and it also illustrated the complex interactions between offsprings’ and parents’ genetic risks for SCZ. Confirming the relevance of childhood trauma, Heins et al. assessed 234 patients with psychosis and 227 healthy controls for the presence of 25 items including emotional, physical and general childhood abuse, and emotional and physical neglect [36]. The OR was 4.53 for childhood trauma in cases versus controls.

Because they confirm that events as long ago as in-utero or infancy, may influence subsequent SCZ, a brief mention is made here about the several prospective studies following birth cohorts that are consistent with an association between viral or bacterial maternal infection and psychiatric disorders in offspring [37], and that are confirmed by animal studies showing maternal immune activation as being sufficient to impart lifelong neuropathology and altered behaviors in offspring [38]. In these prospective studies, the neuropathology includes reduced amounts of MBP, PLP, and myelin, presumably caused by dysfunctional oligodendrocytes. In a major review, Patterson listed 12 reports showing that poly I:C (polyinosinic:polycytidylic acid), which is a synthetic double-stranded RNA used experimentally to simulate viral infections, when injected into pregnant rodents, led to the offspring having multiple behavioral abnormalities, and cerebral abnormalities that included less MBP, PLP, and myelin staining, smaller and denser neurons in the hippocampus, and more pycnotic cells in the cortex [39]. Results of a meta-analysis involving 1035 cases and over 1.2 million controls, suggested that all childhood CNS infections, particularly viral infections, may be associated with a nearly twofold risk of adult SCZ [40].

The preceding two sections described how various stressors cause abnormal oligodendrocytes, and the various molecular mechanisms that are responsible. The present section showed that both prenatal, immediately postnatal, and childhood stressors may apply to at least 33% of SCZ patients [34] and, by inducing abnormalities in myelinating oligodendrocytes with pathogenic consequences for both neural tracts and synaptic function, be antecedents of later SCZ. The term, latency, describes that subsequent to those stressors, SCZ may not become manifest for two or more decades. The following section is a very brief synopsis of the imaging techniques that identify myelin.

### Imaging and myelination

Myelinating oligodendrocytes have many branched processes that wrap around naked axons and cover them with sheaths of myelin in multiple layers. Water that is trapped between those many layers, accounts for ~40% of myelin’s weight. Myelin’s integrity may be assessed post-mortem by electron microscopy. In-vivo, diffusion tensor (tensor refers to scalar values constituting a vector) imaging (DTI) determines the diffusion rate of tissue water; its main application is the imaging of white matter, where the orientation, location, and anisotropy of the tracts can be measured. Anisotropy refers to the non-random movement of water, and FA assesses the degree of anisotropy and quantifies the myelin water because the T2-weighted signal decays faster in the trapped water than in the freely moving water of the intracellular and extracellular spaces. Other DTI measures besides FA include mean diffusivity (MD), which measures the rate of water diffusion; radial diffusivity (RD), which measures diffusion perpendicular to the axon (i.e., largely within myelin); and axial diffusivity, which measures diffusion parallel to the axon. The magnetization transfer ratio (MTR) reflects myelin content by assessing the contrast resulting from H + protons being in three states: bound to macromolecules, in free water, or (the measure of interest) as water between the layers of myelin.

With decreased myelination, FA and MTR decrease, whereas both RD and MD increase. In SCZ and in patients at high risk for subsequent SCZ, multiple studies (see below) have demonstrated increased MD or RD, and decreased FA or MTR.

### Myelin in SCZ is abnormal

Several reports showed myelination defects in SCZ. Among the first, was by Flynn et al. who compared 30 SCZ patients with 27 controls and saw 12% lower water fraction in the patients, uncorrelated with age or years of education [41]. In 19 SCZ patients, Koch et al. saw increased RD that was negatively correlated with activation in the fronto-striato-cingulate network [42]. In 23 SCZ patients, Du et al found that significantly reduced MTR correlated with more adverse outcomes [43]. The largest study was by Zeng et al. in 55 drug-naïve patients with first episodes of SCZ [44]. The patients had lower FA and higher MD; and when 25 of the patients were re-studied after 8 weeks of anti-psychosis medication, the change in FA in the left superior longitudinal fasciculus correlated with the changes in positive symptoms and processing speed (respectively, *r* = 0.56 and 0.47). Palaniyappan et al performed DTI and MTR in 17 SCZ patients and also found a correlation between the reduction in FA and processing speed [45].

### Studies in persons at high risk for SCZ show that in the latent interval there are both premonitory symptoms and abnormalities of neural tracts

If the stated hypothesis were correct, one would expect that there might be premonitory symptoms during the latent interval, when abnormal neural tracts were starting to appear but insufficiently-so for a SCZ diagnosis. Individuals considered to be at high risk for eventual SCZ, form the ideal groups in which to demonstrate if there are premonitory symptoms and structural brain changes during the latent interval.

With regard to premonitory symptoms, Niemi et al. reviewed 16 published studies, with the criteria that the definition of high-risk status was based on having a biological relative with SCZ, and that at least 20 subjects were followed [46]. Ten of the studies followed the subjects for 10 or more years, three of them for 20 or more years. The 16 studies involved 1390 high risk cases and 1432 controls. Niemi et al. summarized the excessive abnormalities seen in the high risk cases as compared with the controls during four stages of childhood and adolescence: between ages 0 and 2, low communicative competence; between ages 2 and 7, anxiety and hostility, more isolated and lonely, disturbed behavior, impulsivity, distractibility, emotional lability; between ages 8 and 12, disturbing school behavior, problems in interpersonal relations, social isolation, poor affective control, more psychopathology, tendency to anhedonia, depression, aggression; between ages 13 and 19, disturbed behavior, poor peer relations, poor social competence, more anxious, greater affective flattening. The following is a direct quote from their article. “Factors which appear to predict SCZ include problems in motor and neurological development, deficits in attention and verbal short-term memory, poor social competence, positive formal thought disorder-like symptoms, higher scores on psychosis-related scales in the MMPI, and severe instability of early rearing environment”. Although subsequently there have been many reports of individuals at high risk for SCZ, they have all reported symptoms that occurred between only 1 and 4 years before the transition to psychosis. As two examples, Kang-Yi et al. found that one or more psychiatric diagnoses had been made during the year before their first diagnosis in 1459 adolescents aged 9–17 years who had been diagnosed with SCZ [47]; and DeVylder et al. reported a 2.5 years’ study in which the presence of baseline, disorganized communication was significantly predictive of the transition to SCZ that was made by 26 of the 100 participants [48]. The report by Niemi et al. substantiates the hypothesis suggested in the present article by showing that symptomatology was spread-out during the long years of latency between infancy and the onset of SCZ.

Since cognition may be abnormal in established SCZ, it might be affected during the latent interval. Fusar-Poli et al. analyzed 19 reports involving a total of 1188 subjects at high risk for SCZ and 1029 controls [49]. The subjects at high risk were impaired relative to controls on tests of general intelligence, executive function, verbal and visual memory, verbal fluency, attention and working memory, and social cognition. Those subjects who later developed psychosis, had even more marked deficits in the verbal fluency and memory domains.

It is also the case that abnormalities of neural tracts might be detectable in the persons at high risk for subsequent SCZ. Observations by Witthaus et al. associated abnormal behaviors with MRI changes [50]. They saw 30 persons in whom at least one of the following occurred several times weekly: ideas of reference; odd beliefs or magical thinking; unusual perceptual experiences; odd thinking and speech; suspiciousness or paranoid ideation; odd behavior or appearance. MRI scans showed diminished white matter volume of the right superior temporal lobe, reflecting abnormal function of myelinating oligodendrocytes. Using the Risky Families Questionnaire [51], Poletti et al. found that among non-psychotic individuals, those with more severe, adverse childhood experiences, putting them at risk for subsequent SCZ, had decreased FA and increased MD, showing more abnormal white matter tracts, affecting the corona radiata, thalamic radiations, corpus callosum, cingulum bundle, superior longitudinal fasciculus, inferior fronto-occipital fasciculus, and uncinate fasciculus [52].

Between them Samartzis et al. and Smieskova et al. summarized the results of brain imaging reported in 12 separate studies of persons at high risk for SCZ [53, 54]. Most often, the observed changes were reduced FA and/or increased MD in frontal and temporal lobe cortices. There were, however, several inconsistencies in the reported results: one study showed reduction in temporal white matter but an increase in left medial temporal white matter in those who transitioned to psychosis versus those who did not; another showed decreased FA in the posterior cingulate white matter but increased in the anterior cingulate; and despite most studies showing decreased gray matter volume in frontal and temporal cortices, it was increased in the cuneus gyrus. There are several possible explanations for these inconsistencies. There may be tissue variation within a brain region that is caused by differences in cell densities, composition of the neuropil, or degrees of myelination of neurons. Another reason may be that there is a variety of inputs to SCZ, both genetic and environmental, and that each input has a different effect on oligodendrocyte function (see ref [55]). Inconsistencies concerning which tracts are affected and whether tract volumes are decreased or increased, could also result from SCZ and its antecedents not being homogeneous but a mélange of different conditions with different causal inputs having different effects. Technical issues involving production and interpretation of the images, may also contribute. However, whatever the explanation, the important finding relative to the current topic, is that there are neural tract changes for as long as 20 years before clinical SCZ obtains.

### What percentage of oligodendrocytes is abnormal in schizophrenia?

The percentage of abnormal oligodendrocytes that persists into adulthood is a question that is unanswerable except by a guess. It cannot be100% because were it so then SCZ would be accompanied by widespread neurologic abnormalities and the disease would appear at an extremely early age. If only a small percentage of oligodendrocytes were abnormal then SCZ would have a very late onset; and if a high percentage were abnormal then SCZ would have a very early onset. Since ~10% of SCZ has either a very early or a very late onset, it might follow that ~10% of schizophrenic patients have abnormal oligodendrocytes that are either a high or low percentage of the total. A report by Bennett et al. confirms that a mixture of normal and abnormal oligodendrocytes may occur [56]. They exposed pregnant guinea pigs to a stressor and found that offspring at term had a 40% decrease in expression of mature oligodendrocytes as shown by decreased levels of MBP, which increased to normal levels by post-natal day 21; it is a reasonable presumption that in a small percentage the abnormal, myelinating oligodendrocytes that had been present at term, would continue into further adult life.

### Ways by which clones of abnormal oligodendrocytes might create abnormal neural tracts

Myelin affects neuronal network formation and function, in several ways, which is why abnormal oligodendrocytes may be central to causing neuropathology. One way is through genetic mechanisms. An intrinsic genetic program directs where and when oligodendrocyte precursors proliferate, migrate and differentiate. Sun L et al. showed that transcription factor EB (TFEB) is highly expressed by differentiating oligodendrocytes and that its loss caused precocious and ectopic myelination in many parts of the murine brain [57]. Another way is through the regulation of Ca^2+^ in oligodendrocytes, which may be through ligand-operated channels, through voltage-operated Ca^2+^ channels, or via Ca^2+^ channels in the endoplasmic reticulum. Responses to glutamate, neuronal potassium, and GABA, also influence OPC, and if intrinsically defective would affect myelination. Finally, small perturbations in timing, i.e., in their conduction speed, may desynchronize the timing of spikes arriving at key synapses and, therefore, affect the functional integrity of neural tracts. Correct conduction speed depends on a relationship between the axon diameter and the thickness and spacing of myelin; there is a reciprocity in this relationship because the proliferation and differentiation of myelin-forming oligodendrocytes is itself regulated by neural activity [58, 59]. Importantly, oligodendrocytes may sense the activity of axons with a wider spatial resolution than a synapse. Thus, Wake et al. showed that release of glutamate from synaptic vesicles present along axons, induced myelin formation, presumably via activation of NMDARs on oligodendrocytes [59].

## Possible weaknesses in the hypothesis that dysfunctional oligodendrocytes account for latency in some patients with schizophrenia

While a role for oligodendrocytes in SCZ has been known for several decades, the new concept suggested here is that oligodendrocytes might account for the long latency between the earliest events in the pathogenesis of SCZ and its clinical manifestations. Potential concerns with the hypothesis and responses to those concerns, are as follows.

First, it could be expected that the dysfunctional oligodendrocytes might cause generalized abnormalities of neural tracts that should then produce diffusely abnormal, neurological physical signs. In fact, neurological soft signs are seen in SCZ, both treated and untreated [60, 61]. Evidence shows, however, that the distribution of oligodendrocytes differs among the regions of the brain. In SCZ patients as compared with controls, Hof et al. saw a 28% decrease of oligodendrocytes in gray matter, cortical layer III, and a 27% decrease of oligodendrocytes in the white matter, which would excessively affect myelination in those particular regions [62]. After their recovery from cuprizone-induced demyelination, rodents had significantly more oligodendrocytes in the anterior part of the corpus callosum compared to more posterior areas [63, 64]. Thus, abnormalities of neural tracts would not be generalized but selective.

Second, the timing of when the neuropathology began may be difficult to ascertain but in many patients the timing of either maternal or perinatal infection, or of childhood abuse, may be established with some confidence, and it is to such patients that this hypothesis primarily applies.

Third, disturbed myelination mainly affects nerve conduction velocity (CV) and not necessarily the anatomy of the neural pathways themselves. Even so, small aberrancies in CV may have profound effects upon the function of neural tracts. Importantly, in a number of ways oligodendrocytes are also involved in synaptic function. One being by the expression of Kir4.1 that contributes to clearance of K + from the peri-axonal space to allow membrane repolarization after its release during action potential firing [65]. Another way is via activity-dependent BDNF signaling, which is involved in synaptic transmission by determining the release of presynaptic glutamate vesicles [66]. Neuronal action potentials also release glutamate, which activates NMDA receptors on oligodendrocytes and NG2 + cells [67]. Next, oligodendrocytes affect synaptic function via intracellular calcium signaling because they have a calcium-sensing receptor [68], so that changes in Ca^2+^ distribution within oligodendrocytes can regulate synaptic mechanisms. Several large-scale genome-wide association studies show a strong and consistent association between susceptibility for SCZ and a SNP, rs1006737, that is related to the L-type voltage gated calcium channel (VGCC) subunit gene *CACNA1C* [69]. At synapses, VGCCs form large signaling complexes in the presynaptic nerve terminal, which are responsible for the calcium entry that triggers neurotransmitter release and short-term presynaptic plasticity [70]. VGCCs in synaptic spines and dendritic shafts, also affect post synaptic stability [71]. Finally, oligodendrocytes affect axonal CV in ways other than by myelination; human NMDAR-specific monoclonal antibodies specifically alter the function of the NMDARs in oligodendrocytes, causing a decreased expression of GLUT1, which is required for normal axonal function [72].

### Potential therapies for schizophrenia in patients whose history includes maternal infection during pregnancy and infantile or childhood trauma

Fingolimod and siponimod will be the main therapies discussed because of their well-documented benefits to oligodendrocytes and neural tracts.

Fingolimod has been mostly used in multiple sclerosis (MS), and reports show that it benefits neural tracts, presumably via myelinating oligodendrocytes. In 25 subjects, Gurevich et al. showed that in MS, those having impaired pyramidal function at baseline, fingolimod induced a significant increase in FA (*P* = 0.002) and decrease in RD (*P* = 0.03) in the corticospinal tract [73]. In another MS study, Saraste also showed that fingolimod improved FA and decreased RD in the cingulate (*p* = 0.027 and 0.041, respectively [74]. In experimental allergic encephalitis (EAE) in mice, Zhang et al. scored neurological function on the basis of diminished tail tone, ataxia, paresis of hind limbs or forelimbs, or tetra-paresis [75]. Fingolimod increased the OPCs, which led to an increased area of myelination and improved neurological function. Miron et al. administered fingolimod during the 4–6 weeks after initiation of EAE; it decreased paralysis and normalized neurologic function, reversed BBB permeability, and reduced demyelination [76]. Miron et al. also demyelinated cultured slices of hind brain and cerebellum by adding lysolecithin; they then added fingolimod to the cultures for 2 weeks, which led to production of mature oligodendrocytes and many myelin sheaths [76]. Gol et al. induced myelin damage in the hippocampus and cerebral cortex by treating mice with pentylenetetrazol; fingolimod protected against demyelination in the CA1 and CA3 hippocampal regions and significantly increased neuronal cell number [77].

Fingolimod also benefits synaptic function and dendritic spine density, which reflects synaptic number. Using cultures of healthy, mature primary hippocampal neurons, Patnaik et al. showed that fingolimod regulated the architecture of dendritic spines and induced an ~10% increase in their density [78]. In EAE, Rossi et al. saw that fingolimod markedly decreased the loss of dendritic spines as well as causing a reversal of both pre-synaptic hyperactive NMDARs and post-synaptic hypersensitive AMPARs [79]. This confirmed the earlier findings of Sim-Selley et al., that S1P receptors, whose actions are mimicked by fingolimod, are prevalent throughout the cerebral cortex and amygdala in rats, and that their activation inhibited, beneficially, glutamatergic neurotransmission [80]. In 13 MS patients, Landi et al. applied trans-magnetic stimulation (TMS) to the motor cortex at baseline before starting a daily dose of fingolimod [81]. Measurements at baseline and then 60 days later, showed that fingolimod caused a significant reduction in the stimulatory effect of TMS, which is mainly mediated by glutamate.

But it is also the case that in SCZ brains there is an element of inflammation [82]; and, of course, fingolimod’s benefit for MS largely results from its anti-inflammatory effect. In SCZ, Francis et al. showed that fingolimod caused significant reductions in peripheral blood (PB) lymphocytes and that there were correlations (all *r* = −0.42) between FA increases in the corpus callosum and superior longitudinal fasciculus and the percent decrease in PB lymphocytes [83].

Thus, in interpreting the results, is the benefit to SCZ from anti-inflammation per se, from enhanced myelination caused by oligodendrocytes, or both? That could be elucidated by a clinical trial having two groups of patients: in one group, there would be evidence of either maternal inflammation or infantile trauma; and the other group would be selected for having had neither of those events. The first group would be predicted to show increased FA and reduced RD, and the second group would be predicted to show neither.

Siponimod, however, is another agonist of S1PR1&5. It has high affinity for the S1P5 receptors that are present on oligodendrocytes in all stages of its development; and it decreased oligodendrocyte cell death and axon demyelination in a mouse model of MS [84].

Crucial to the question whether its anti-inflammatory effect is what benefits the brain, Gentile et al. reported that siponimod’s effect on EAE, is independent of peripheral lymphocytopenia; the highest dose that they used completely inhibited EAE development independently of minimal PB lymphocytopenia [85]. This finding suggests that the benefit of S1P activation is not due to its anti-inflammatory effects. Further important support that reduced brain inflammation is not the reason for neural tract benefit from S1PR1&5 agonism, was provided by Hundehege et al., who found that intracerebral siponimod treatment did not reduce immune cell infiltration into the brain parenchyma compared to non-treated controls: CD45 + , CD4 + , and CD8 + cell numbers were unchanged [86]. The effect of fingolimod has not been studied in this way but there is no reason to suppose that it would be different from the effect of siponimod.

### Adverse events from potential therapies

Potential adverse events (AE) of therapy are always a concern. They may be minimized by using reduced doses of fingolimod, or titration of initial doses of siponimod. For fingolimod, pooled data from three studies (*n* = 1640) showed that on treatment initiation, fingolimod 0.5 mg was associated with transient but seldom (0.5%) symptomatic, bradycardia and second-degree atrioventricular block, minor blood pressure increases, generally asymptomatic liver enzyme elevations (9%), and macular oedema (0.4%) [87]. Fingolimod in higher dose (1.25 mg, *n* = 1776) gave more frequent AEs/SAEs. So, if used in patients with SCZ, fingolimod dosage should be no higher than 0.25 mgs daily; and before starting treatment ophthalmological examination and EKG must be performed.

When siponimod treatment is initiated, bradycardia can be avoided by a dose titration such that at low dosages there is down-regulation of S1PR and therefore minimal effects on heart rate once the full dose is achieved. Selmaj et al. saw a dose-dependent decrease in heart rate on treatment initiation in a study that did not use a dose titration [88]; but in a dose-blinded extension of the same study, Kappos et al. reported reduced AEs when dose titration had been used at initiation of siponimod [89]. A total of 184 participants received siponimod at doses of either 10, 2, 1.25, 0.5, or 0.25 mg daily, according to a dose-titration schedule (siponimod, 0.25 mg, on day 1 escalating over 10 days to the assigned dose). Only two patients (1.1%) had second-degree atrioventricular block, that was transient and not considered clinically significant. The most common AEs were nasopharyngitis, insomnia, headache, lymphopenia, and increased alanine aminotransferase. There were no cases of macular edema.; nor were there severe or systemic opportunistic infections in any treatment group. Thus, if a clinical trial were undertaken to validate the hypothesis that in selected subjects the symptoms of SCZ might improve from administration of an S1P agonist, using siponimod might be preferred over fingolimod.

## Discussion

Discussion about the effects of oligodendrocytes in SCZ have been provided by others--notably by Takahashi et al. [90] and Chew et al. [91]. What is different here is the hypothesis that abnormalities in oligodendrocyte function may sometimes account for the mystery of the long latency. In an article whose subtitle was, ‘Findings that every theory of SCZ should explain’, MacDonald and Schultz distilled the science of SCZ into 22 facts [92]. The present article is solely an attempt to account for just one of those 22 facts. The duration of latency, that may last for as long as 20 years in the commoner early-onset disease, or for 40+ years in the late-onset variant, is rare in pathology with very few exceptions: post radiation cancer may have a multiyear latency and so may type 2 diabetes (T2DM); but even in T2DM, any premonitory symptoms are during a very few years before the disease emerges, not 20 and certainly not 40+ years before then. Relevant to the latency in SCZ, are the human and animal studies that followed-up birth cohorts showing an association between viral or bacterial, maternal infection and psychiatric disorders and lifelong neuropathology in offspring [37, 38]. Also relevant, injections of the inflammatory cytokine IL-1β into 15–30 days’ post-natal mice, produced a block in the maturation of oligodendrocytes, reductions in levels of myelin proteins (MBP, PLP, and MAG), increased numbers of non-myelinated neurons, axonopathy, and long-lasting behavioral changes [93].

If the hypothesis is correct, the possibility follows of using treatment to address the abnormal oligodendrocytes. Interestingly, some anti-psychosis drugs, e.g., quetiapine, olanzapine, and haloperidol, promote NG2 maturation, oligodendrocyte regeneration, and myelin repair [94]. However, in this article, fingolimod and siponimod were the suggested potential therapies because of their very well-documented benefits to oligodendrocytes and neural tracts; and the evidence presented, shows that, via improved function of oligodendrocytes, fingolimod and siponimod induced repair of damaged neural tracts such as are seen in SCZ.

### Summary

The hypothesis is proposed, that oligodendrocytes with abnormal function, explain the latency between onset of the cerebral neuropathology and clinical presentation in those SCZ patients affected by inciting elements decades before the presentation of the clinical syndrome. Data show that one of the several functional abnormalities is, via several molecular pathways, blockage of oligodendrocyte maturation to myelin-forming cells. Fingolimod and siponimod, drugs that improve the function of oligodendrocytes also improve neurological function. Clinical trial would validate the potential benefit from fingolimod or siponimod in appropriate patients with SCZ and, concurrently, would validate the hypothesis.
